# Growth Dynamics of Patient-Provider Internet Communication: Trend Analysis Using the Health Information National Trends Survey (2003 to 2013)

**DOI:** 10.2196/jmir.7851

**Published:** 2018-03-29

**Authors:** Will L Tarver, Terri Menser, Bradford W Hesse, Tyler J Johnson, Ellen Beckjord, Eric W Ford, Timothy R Huerta

**Affiliations:** ^1^ Health Services Research and Development Service Center for Health Information and Communication Richard L Roudebush VA Medical Center Indianapolis, IN United States; ^2^ Center for Outcomes Research Houston Methodist Research Institute Houston, TX United States; ^3^ Health Communication and Informatics Research Branch National Cancer Institute Bethesda, MD United States; ^4^ Department of Family Medicine The Ohio State University Columbus, OH United States; ^5^ Population Health Program Design and Engagement Optimization UPMC Health Plan Pittsburgh, PA United States; ^6^ Department of Health Care Organization and Policy University of Alabama at Birmingham Birmingham, AL United States

**Keywords:** internet, health communication

## Abstract

**Background:**

Communication is key in chronic disease management, and the internet has altered the manner in which patients and providers can exchange information. Adoption of secure messaging differs among patients due to the digital divide that keeps some populations from having effective access to online resources.

**Objective:**

This study aimed to examine the current state of online patient-provider communication, exploring trends over time in the use of online patient-provider communication tools.

**Methods:**

A 3-part analytic process was used to study the following: (1) reanalysis, (2) close replication across years, and (3) trend analysis extension. During the reanalysis stage, the publicly available Health Information National Trends Survey (HINTS) 1 and 2 data were used with the goal of identifying the precise analytic methodology used in a prior study, published in 2007. The original analysis was extended to add 3 additional data years (ie, 2008, 2011, and 2013) using the original analytical approach with the purpose of identifying trends over time. Multivariate logistic regression was used to analyze pooled data across all years, with year as an added predictor, in addition to a model for each individual data year.

**Results:**

The odds of internet users to communicate online with health care providers was significantly and increasingly higher year-over-year, starting in 2003 (2005: odds ratio [OR] 1.31, 95% CI 1.03-1.68; 2008: OR 2.14, 95% CI 1.76-2.59; 2011: OR 2.92, 95% CI 2.33-3.66; and 2013: OR 5.77; 95% CI 4.62-7.20). Statistically significant socio-economic factors found to be associated with internet users communicating online with providers included age, having health insurance, having a history of cancer, and living in an urban area of residence.

**Conclusions:**

The proportion of internet users communicating online with their health care providers has significantly increased since 2003. Although these trends are encouraging, access challenges still exist for some groups, potentially giving rise to a new set of health disparities related to communication.

## Introduction

Effective communication between a patient and their health care provider is central to the provision of medical care [1-3]. Without effective communication, and the trust it can build, chronic disease management can be tenuous and the delivery of high quality care is impaired [4], medication adherence is reduced, and patients rely on low-quality information for making decisions that affect their health [5]. The internet has transformed the way people communicate, providing alternative means of communication (eg, email, secure messaging, instant messaging, and online videos) that supplement or, in some instances, have replaced the traditional in-person and telephonic communications. These alternative communication channels are being utilized with greater frequency and are becoming a normal part of service delivery in the health care industry. Empirical studies have found a positive correlation between increased electronic communication and health outcomes.

The use of internet-enabled communication can facilitate patient engagement and create better documentation modes for patient-provider communication [6,7]. There is a growing interest in shifting care processes (eg, requests for referral, test results) to technology-enabled models. For physicians, decreasing face-to-face consultation time for low-value administrative activities allows them to focus on more important clinical encounters [6,7]. For patients, the new communication channels can reduce access burdens related to navigating the care system, including the transaction costs associated with seeking care (eg, transportation, taking time off of work to seek health care). A recent study by Reed and colleagues [8] found that patients with higher out-of-pocket costs were significantly more likely to use secure email as their first method of contact with respect to their health care. In addition, many patients who used a secure email to communicate with their health care providers reported that it reduced their phone contacts and/or office visits.

The use of electronic communication with patients provides important opportunities for the provision of patient-centered care [9]. Electronic communication has been found to improve patient satisfaction and saves patient time. However, providers who use electronic communication extensively with their patients have noted there is a tradeoff that needs to occur if efficiency gains are to be realized. In particular, extensive email communication increases doctors’ workload unless the number of office visits per patient is reduced [10]. A systematic review of patient-provider email communication found the benefits of electronic communication to be recognized by patients and providers alike (eg, ease of communication and ability to improve health care), but several included studies also identified barriers to its use including workload and time demands, confidentiality and security, lack of reimbursement, and inappropriate use of email by patients [11]. One policy change these studies suggest is that insurers should reimburse for electronic communication to promote online patient engagement. Electronic communication is an avenue that may be able to effectively address basic patient questions, leaving the patient visit to focus on more critical issues and concerns.

Despite early literature showing that patients were receptive to communicating through email with their providers [12], significant challenges to both patients and providers have prevented its widespread use (eg, the digital divide, referring to the lack of equity in availability of technologies by demographic characteristics and geographic location [6]; lack of physician reimbursement [6]; and mixed evidence in support of electronic communication on health outcomes [13,14]). In 2007, Beckjord and colleagues [15] used the Health Information National Trends Survey (HINTS) in a benchmark study reporting the prevalence of, and factors related to, use of online communication between patients and their providers. The study found a low prevalence of online patient-provider communication, which significantly increased, from 7% of internet users to 10% between the years 2003 and 2005. In addition, factors that influenced online communication among internet users included higher education, living in a metro area, having poorer health status, and having a personal history of cancer. Since this publication, the growth of the internet has changed the landscape of how people search for and receive messages regarding health and their health status (health information seeking) [16] and how they communicate electronically within an online format (online communication) [17] in the health arena. For example, the internet is used to gather health information by 66% of adults with no chronic conditions and 51% of those with chronic conditions [18]. Although overall access to the Web has increased greatly, with 84% of American adults using the internet in 2015, an increase of nearly 65% from 2000 when 51% of the population used the internet [19]; the digital divide continues to be an issue. Home internet availability is lower among older populations, racial or ethnic minorities, less educated groups, those with lower incomes, and for people that reside outside of metropolitan areas [20-22]. However, increased internet access has also been seen in the underserved populations through the proliferation of mobile devices, which make the internet more accessible [22]. The widespread use of mobile devices has also influenced the way people communicate. For example, electronic communications such as text messaging and email are being used by more than 90% of the population [23].

The Diffusion of Innovation Theory discusses the process by which a new product or technology is adopted by a given population [24]. Understanding the diffusion of online communication can inform the ways in which providers interact with patients. Over half of US health care providers (57%) reported having a patient portal in place in 2012 [25]. The adoption of patient portals has been encouraged by federal policy initiatives such as Meaningful Use; a core objective for Stage 2 Meaningful Use is to use secure electronic messaging to communicate with patients on relevant health information to impact patient care and safety [26]. Kannry and colleagues noted that there is an opportunity to increase secure messaging, citing Ralston and colleagues’ study, which indicated that nearly a third of outpatient encounters could be conducted via secure messaging [27,28]. New, anecdotal evidence suggests that the use of patient portals for communication can help lower overhead costs by reducing the patient call volume and decreasing the overall time required to communicate with patients [29]. Despite these technological advancements, little is known about the current state of online patient-provider communication and how its use has changed over time. Such data will be valuable in informing us about the future direction of online patient-provider communication.

The objectives of this paper were to (1) reproduce and replicate the initial study conducted by Beckjord and colleagues [15], (2) examine the current state of online patient-provider communication, and (3) explore trends over time in the use of online patient-provider communication. We expand on the original study by utilizing data from 5 iterations of the HINTS dataset to explore whether online patient-provider communication and the sociodemographic factors influencing its use have changed over time. Samples were weighted to make the samples representative of the US population.

## Methods

### Data

Although HINTS is a dataset that is maintained by National Cancer Institute and does include several questions that focus on cancer, the population surveyed is random and many of the questions relate to general health communication. HINTS has 6 iterations, 5 of which include the requisite variables to extend Beckjord and colleagues’ study. The first iteration of HINTS (HINTS 1) was conducted from October 2002 through April 2003. Data collection was achieved via a random digit dial telephone survey, which generates phone numbers at random. Selected phone numbers were submitted to a telephone matching service in an effort to remove nonresidential numbers. A computer-assisted telephone interview (CATI) format was utilized to accommodate complex skip-patterns. Survey administration averaged 30 min per respondent, and data were collected from 6369 respondents.

HINTS 2 (2005) data were collected from February 2005 through August 2005. Like HINTS 1, HINTS 2 also used a random digit dialing telephone survey with a CATI format. All data collection procedures were identical to HINTS 1. Data were collected from 5586 respondents.

The HINTS 3 (2008) data were collected from January through May 2008. The HINTS 3 sample design included 2 data collection methods. One sample was drawn as a random digit dialing telephone survey, using a CATI format. The second national random sample was selected from a list of addresses from the United States Postal Service (USPS) administrative records. Data were collected from 4092 respondents via CATI and from 3582 respondents via mail. Differences between the CATI and USPS samples were tested. Despite the differences between the 2 populations in education, income, general health status, metropolitan statistical area, and internet use, all data were included to maintain fidelity to the original study.

HINTS 4, Cycles 1 and 3 data were collected from October 2011 through February 2012 and September through December 2013, respectively. The sample design for both surveys consisted of a single-mode mail survey, using the Next Birthday Method for respondent selection. The sample design for the interview portion of the survey consisted of 2 stages. In the first stage, a stratified sample of addresses was selected from a file of residential addresses. In the second stage, 1 adult was selected within each sampled household. The sampling frame consisted of a database of addresses used by Marketing Systems Group to provide random samples of addresses. Complete data were collected from 3959 respondents in 2011 and from 3185 respondents in 2013.

### Measures

Sociodemographic characteristics included age (18-34, 35-49, 50-65, or >65 years), sex (male or female), education (less than high school, high school graduate, some college, or college graduate), income (<US $10,000; US $10,000-14,999; US $15,000-19,999; US $20,000-34,999; US $35,000-49,999; US $50,000-74,999; or ≥US $75,000), race and ethnicity (white, Hispanic, black, or Asian, other), metropolitan area (metro or non-metro), and health insurance status (yes or no), one’s own perceived health status (excellent, very good, good, fair, or poor), and cancer history (yes or no). Measures relating to use of technology and electronic communication were assessed in the form of questions, which asked whether they used the internet (yes or no) and whether respondents had communicated online with a provider in the past 12 months (yes or no).

There were differences in how data were categorized for the income and perceived health status variables. The categories for the income variable in HINTS changed over time, and the income categories used in this study of trends over time reflect that. Additionally, the categories for perceived health status remained stratified for a more in-depth analysis, and were not dichotomized as in the original study.

### Analytic Framework

A 3-part analytic process was used to study (1) reanalysis, (2) close replication across years, and (3) trend analysis extension (see Multimedia Appendix 1). During the reanalysis stage, the publicly available HINTS 1 and 2 data were used with the goal of identifying the precise analytic methodology used in the original 2007 paper. This stage served as a precursor to replication, ensuring that the model with additional data years is done with a high degree of fidelity to the analytic framework established as valid in the literature [30]. In the close replication stage, we made minor alterations to the original analytical model to enable effective analysis of multiple data years in a manner that is as close to the original model as possible, while taking into account all available variables in each data year. Finally, we engaged in extension, that is, the analysis of additional data years, 2008, 2011, and 2013, using the same analytical approach with the purpose of identifying trends over time. The first author of the original study was invited to join the study at this stage.

### Statistical Analyses

Analyses were conducted using both unweighted and weighted data. All weighted analyses were conducted using jackknife variance estimation to account for the complex survey design and provide nationally representative population estimates. Mirroring the original paper [15], respondents’ sociodemographic characteristics were calculated by percentage of internet users for the years 2003, 2005, 2008, 2011, and 2013 (Table 1).

The same characteristics were then calculated by the percentage of patients who communicated online with a provider (Table 2). We employed a weighted, multivariate logistic regression for each individual data year (Multimedia Appendix 2), as well as an additional model, which pooled the data across years (Table 3) and contained the year as a predictor variable to identify potential predictors of online communication with a health care provider. Each model was adjusted to control for all sociodemographic characteristics and assessed online communication with a provider where responses of “Yes” represented a positive outcome. The original study contained a table of the results of the bivariate year-specific analyses, but trying to assess changing trends over time related to demographic factors may present spurious correlations as associations; thus, we omitted this approach in this study.

## Results

### Sample Characteristics and Bivariate Analyses

The sample characteristics of internet users for the reproduced Beckjord and colleagues [15] study using HINTS 2003 and 2005 data, and extending it to include the 2008, 2011, and 2013 HINTS iterations are presented in Table 1.

In 2003, 7% of internet users reported communicating online with a health care provider in the past 12 months, increasing to nearly 10% of internet users in 2005. These estimates are consistent with the original study, and the percentage of internet users reported communicating online with health care providers increased to 14% in 2008, 19% in 2011, and 30% in 2013.

Consistent with Beckjord and colleagues’ original study, individuals who communicated online with health care providers had significantly more years of education and were more likely to reside in a metropolitan area in 2003. In 2005, these individuals were more likely to be female and were more likely to have a personal history of cancer. The reproduction of the original study found minor differences in the means of some variables, which may be attributed to variations in the SUDAAN (Research Triangle Park, North Carolina) and STATA (College Station, Texas) jackknife algorithms. In addition, we found 2 differences between sociodemographic variables and communicating online with health care providers for 2005 when comparing results with those from the original study (see Table 2). These 2 differences were (1) communicating online with health care providers was not found to be statistically significantly associated with more years of education (*P*>.05) and (2) annual income was statistically significant (*P=*.038).

### Multivariate Analyses

The findings of our multivariate models conflicted with the original study [15] in 2 instances in 2005. First, gender was not found to be statistically significant, and second, we found that individuals who reported poor health had higher odds of communicating online with health care providers, which was not reported in the original study. Notably, several variables were consistently found significant across multiple years. For example, gender was statistically significant for the years 2011 and 2013; having a history of cancer was statistically significant for 2005 and 2013; and geographic location was statistically significant for 2003, 2008, and 2011 (all *P* values were <.05). Full results of multivariate analyses by HINTS year is provided in Multimedia Appendix 2.

When exploring trends in the use of online communication with health care providers, we found a significant increase between the years 2003 and 2013. More specifically, as compared with 2003, the odds of internet users to communicate online with health care providers was found to be significantly and increasingly higher in the subsequent years, 2005 (odds ratio [OR] 1.31, 95% CI 1.03-1.68, *P=*.027), 2008 (OR 2.14, 95% CI 1.76-2.59, *P<*.001), 2011 (OR 2.92, 95% CI 2.33-3.66, *P<*.001), and 2013 (OR 5.77, 95% CI 4.62-7.21, *P<*.001).

When looking at all 5 years of HINTS data and adding in year as a covariate, the odds of communicating with a provider via the internet increased for women (OR 1.31, 95% CI 1.10-1.55, *P*=.002), college graduates (OR 1.88, 95% CI 1.08-3.28, *P*=.026), and data year (see Table 3). Conversely, the odds of communicating with a provider via the internet decreased for (1) individuals between 65 and 74 years of age (OR 0.70, 95% CI 0.52-0.94, *P*=.018), (2) the uninsured (OR 0.59, 95% CI 0.41-0.85, *P*=.005), (3) individuals with no history of cancer (OR 0.68, 95% CI 0.54-0.84, *P*=.001), and (4) individuals living in a nonmetro area (OR 0.63, 95% CI 0.49-0.80, *P*<.001).

**Table 1:** Weighted percentage of Health Information National Trends Survey (HINTS) internet users.

Characteristic		HINTS 2003 (n=3982), %	HINTS 2005 (n=3244), %	HINTS 2008 (n=5078), %	HINTS 2011 (n=2914), %	HINTS 2013 (n=2284), %
Communicated online with a provider in the past 12 months		7.00	9.63	13.57	19.11	29.70
**Age, years**						
	18-34	38.29	37.74	36.16	34.89	31.36
	35-49	35.77	33.25	32.81	30.33	32.63
	50-64	19.82	22.09	23.03	24.67	25.23
	65-74	4.37	4.97	5.49	6.99	7.13
	75 or older	1.76	1.95	2.51	3.12	3.64
**Gender**						
	Male	49.50	48.22	47.13	47.81	48.87
**Education**						
	Less than high school	6.84	5.30	5.39	7.25	5.14
	High school graduate	25.51	23.58	21.76	18.86	19.60
	Some college	32.98	38.28	40.37	34.45	36.53
	College graduate	34.67	32.84	32.48	39.45	38.73
**Annual income, US $**						
	<10,000	2.76	3.20	4.35	7.78	4.78
	10,000 to <15,000	2.14	3.26	4.50	5.17	4.06
	15,000 to <20,000	4.45	3.12	3.18	6.53	5.27
	20,000 to <35,000	17.45	13.20	12.59	15.51	12.37
	35,000 to <50,000	18.32	13.88	14.38	12.30	15.29
	50,000 to <75,000	22.34	25.52	21.93	18.42	19.71
	75,000 or more	32.53	37.82	39.06	34.29	38.52
**Race/ethnicity**						
	White	78.16	76.57	74.74	68.71	70.36
	Hispanic/Latino	6.91	7.55	9.06	13.68	13.03
	African American	8.71	8.90	9.35	10.34	9.36
	Asian	3.10	3.24	4.96	5.34	4.85
	Other	3.13	3.74	1.90	1.94	2.40
**Health insurance**						
	Yes	89.32	87.50	86.01	82.83	84.56
**Health status**						
	Excellent	15.31	13.35	11.84	15.19	13.98
	Very good	34.24	33.27	40.42	39.56	38.98
	Good	34.62	36.98	36.29	32.80	36.71
	Fair	13.12	13.78	9.70	10.48	9.26
	Poor	2.72	2.62	1.75	1.97	1.07
**History of cancer**						
	Yes	8.42	9.12	5.96	6.98	7.46
**Metropolitan statistical area**						
	Metro area county	84.38	81.79	85.13	84.88	83.24

**Table 2:** Weighted percentage of patients who communicated online with a health care provider. Statistically significant values are italicized. HINTS: Health Information National Trends Survey.

Characteristic		HINTS 2003, %	*P* value	HINTS 2005, %	*P* value	HINTS 2008, %	*P* value	HINTS 2011, %	*P* value	HINTS 2013, %	*P* value
**Age in years**			.11		.66		.19		.26		.16
	18-34	6.36		10.25		12.42		18.13		27.7	
	35-49	6.75		9.27		15.64		17.34		33.13	
	50-64	9.32		10.09		12.94		20.04		30.41	
	65-74	4.62		6.45		14.14		20.82		22.18	
	75 or older	6.72		7.01		11.05		29.38		21.24	
**Gender**			.17		*.03* ^a^		.98		*.02* ^a^		.21
	Male	7.64		7.90		13.62		15.97		27.58	
	Female	6.38		11.24		13.58		22.12		31.87	
**Education**			*<.001* ^b^		.145		*<.001* ^b^		*<.001* ^b^		*<.001* ^b^
	Less than high school	3.17		8.53		10.80		8.18		17.20	
	High school graduate	3.53		6.60		9.74		10.37		15.25	
	Some college	7.28		10.09		11.44		17.52		29.06	
	College graduate	10.31		11.66		19.66		25.74		39.42	
**Annual income (US $)**			.32		*.04* ^a^		*.007* ^b^		*.04* ^a^		*<.001* ^b^
	<$10,000	8.51		16.01		11.68		15.12		17.91	
	$10,000 to <$15,000	9.32		7.02		13.50		9.43		23.86	
	$15,000 to <$20,000	8.97		3.84		10.64		14.04		14.46	
	$20,000 to <$35,000	6.43		7.85		8.71		12.36		13.90	
	$35,000 to <$50,000	5.53		7.57		11.54		17.62		26.58	
	$50,000 to <$75,000	6.51		8.43		12.05		17.75		30.59	
	$75,000 or more	9.16		12.78		19.72		25.36		41.14	
**Race/ethnicity**			.96		.36		.99		.16		.18
	White	7.28		9.52		13.80		19.75		29.55	
	Hispanic/Latino	6.42		5.93		14.15		17.05		29.05	
	African American	6.14		11.33		13.02		12.35		31.74	
	Asian	7.08		11.09		13.94		26.07		45.47	
	Other	7.36		15.59		15.18		30.78		25.21	
**Health insurance**			.56		.30		*.003* ^b^		*.001* ^b^		*.003* ^b^
	Yes	7.25		10.08		14.68		21.27		32.58	
	No	6.26		7.83		6.74		8.54		15.65	
**Health status**			.59		.20		.27		.59		.16
	Excellent	7.57		7.89		16.60		22.39		38.12	
	Very good	6.55		9.83		13.57		17.25		26.59	
	Good	6.83		10.61		12.65		20.73		30.60	
	Fair	8.66		7.81		15.60		15.80		24.49	
	Poor	6.49		17.85		8.58		22.34		31.23	
**History of cancer**			.11		*.01* ^a^		.16		.12		*.04* ^a^
	Yes	9.37		14.60		16.44		23.82		39.88	
	No	6.80		9.14		13.53		18.75		29.01	
**Metropolitan statistical area**			*.002* ^b^		.14		*.002* ^b^		*.001* ^b^		*.002* ^b^
	Metro area county	7.51		10.11		14.20		20.65		31.72	
	Nonmetro area county	4.28		7.46		9.90		10.42		19.56	

^a^*P*<.05.

^b^*P*<.01.

**Table 3:** Odds of communicating online with a health care provider within past 12 months across years (n=14,446). Statistically significant values are italicized. HINTS: Health Information National Trends Survey.

Characteristic		HINTS 2003-2013, odds ratio (95% CI)	*P* value
**Age in years**		Reference: 18-34	
	35-49	0.97 (0.78-1.20)	.80
	50-64	0.93 (0.75-1.17)	.54
	65-74	*0.70 (0.52-0.94)*	*.02* ^a^
	75 or older	1.16 (0.78-1.73)	.47
**Gender**		Reference: male	
	Female	*1.31 (1.10-1.55)*	*.002* ^b^
**Education**		Reference: less than high school	
	High school graduate	0.83 (0.48-1.46)	.52
	Some college	1.43 (0.82-2.49)	.20
	College graduate	*1.88 (1.08-3.28)*	*.03* ^a^
**Annual income (US $)**		Reference: <US $10,000	
	$10,000 to <$15,000	1.17 (0.55-2.49)	.68
	$15,000 to <$20,000	0.84 (0.38-1.86)	.67
	$20,000 to <$35,000	0.87 (0.44-1.71)	.69
	$35,000 to <$50,000	1.17 (0.61-2.25)	.64
	$50,000 to <$75,000	1.27 (0.68-2.38)	.45
	$75,000 or more	1.75 (0.94-3.23)	.08
**Race/ethnicity**		Reference: white	
	Hispanic/ Latino	1.04 (0.77-1.40)	.81
	African American	1.00 (0.75-1.32)	.98
	Asian	1.22 (0.85-1.76)	.29
	Other	1.21 (0.82-1.78)	.34
**Health insurance**		Reference: yes	
	No	*0.59 (0.41-0.85)*	*.005* ^b^
**History of cancer**		Reference: Yes	
	No	*0.68 (0.54-0.84)*	*.001* ^b^
**Metropolitan statistical area**		Reference: metro area county	
	Nonmetro area county	*0.63 (0.49-0.80)*	*<.001* ^b^
**Data year**		Reference: 2003	
	2005	*1.31 (1.03-1.68)*	*.03* ^a^
	2008	*2.14 (1.76-2.59)*	*<.001* ^b^
	2011	*2.92 (2.33-3.66)*	*<.001* ^b^
	2013	*5.77 (4.62-7.20)*	*<.001* ^b^

^a^*P*<.05.

^b^*P*<.01.

## Discussion

### Principal Findings

Beckjord and colleagues [15] reported the prevalence of and changes in the use of online patient-provider communication in 2003 and 2005 and described sociodemographic and health-related factors associated with its use. In this study, we were able to use subsequent iterations of the HINTS dataset to describe the current state of online patient-provider communication and understand how its use has changed over time. The data year is shown to be statistically significant in all years with the odds of communicating with a provider online more than quadrupling from 2003 to 2013, the latter indicative of the diffusion of technology over time. This finding coincided with a recent study showing a significant increase in surgeons’ use of secure messaging; the proportion of outpatient interactions was 5.4% in 2008 compared with 15.3% in 2010 [31].

Although we were able to match the majority of the coefficients in the re-analysis stage, there were some minor differences, which may have been attributable to differences in the jackknife algorithm deployed in the software packages used. That said, the interpretation and findings of the first year (2003) did not change as a result; however, several changes were experienced in the 2005 analysis. These differences may be partially attributed to higher estimates of internet use among low-income individuals in 2005. These estimates are not surprising, given the literature reporting interest among underserved populations in using electronic and internet-based tools to communicate with their providers [32]. In addition, evidence shows increasing adoption of patient portals among providers serving underserved populations in efforts to increase patient engagement [33]. The health disparity literature brings attention to the importance of the quality of patient-provider communication when providing medical care to minority populations [34] and its contribution to disparities in medical care [35,36].

We also found that the use of online patient-provider communication steadily increased over time between the years 2003 and 2013. This upsurge in use may be largely attributed to increased interest in the use of online communication tools [32]. In addition, recent attention has been given to using online communication with providers as a means to provide patient-centered care [37] while federal policy initiatives require that physicians provide the use of secure electronic messaging to their patients through Meaningful Use Stage 2 requirements [27]. This federal push to adopt electronic health records has also led to an increase in the implementation and availability of tools to facilitate communication between patients and their providers, such as personal health records (PHRs). It is projected that 75% of adults will use PHRs by the year 2020 [38].

Beckjord and colleagues [15] also explored sociodemographic and health-related factors associated with the use of online communication with a health care provider. We found that in 2003 and 2005, women were marginally more likely to communicate online with their providers, and significantly more likely to communicate with their providers in 2011 and 2013. This is consistent with findings from a previous study identifying women as being more likely to use email to communicate with their doctors [39]. The trends may be explained by understanding gender differences in use of the internet. According to the Pew Research Center [19], men were more likely than women to use the internet in the early 2000s. However, this gap gradually decreased over the years and became equal around the year 2008. In addition, there is evidence that women are more likely than men to use the internet to access health information and to get support for health problems [40]. A recent study looking at patient portal use found that education and sex remain statistically significant when controlling for internet access and preference of communication mode (in person or over the phone vs patient portal) [41].

Being located in a metropolitan area is also shown as relatively consistent when it comes to communicating online with a health care provider. This may be partially attributed to the digital divide. There is literature that suggests patients located in urban locations are more likely to use electronic messaging to communicate with their providers [39], whereas physicians located in urban areas are more likely to provide secure messaging to their patients [42].Finally, having a history of cancer was also found to be associated with online patient-provider communication. Due to the complexity of cancer care, patients often leave visits with their physicians overwhelmed with information and confused about their condition and treatment [43]. Literature suggests that patients need multi-level communication to facilitate information exchange and foster patient-provider relationships [44]. As a result, cancer patients benefit from an asynchronous platform that allows them to access information and engage with their health care team at their convenience. Although these data do capture general health status and cancer history, they do not include other chronic conditions. We were therefore unable to explore the relationship between chronic illnesses outside of cancer and the use of online communication with a health care provider. However, the literature does suggest that these communication technologies will also help patients to more effectively manage other chronic illnesses such as diabetes and hypertension [45]. The nonsignificant finding for health status, measured by a 5-point Likert scale, may too broadly capture respondents’ health state.

### Limitations

HINTS is subject to the same issues all surveys and self-report instruments are, that is, low response rates, potential sampling bias, social desirability issues particularly around issues of smoking and other lifestyle choices, and item limitations. The HINTS response rate was 33% in 2003, 21% in 2005, 21% for telephone survey and 31% for mail surveys in 2007, 37% in 2011, and 35% in 2015; response rates are not a deterministic indicator of bias [46]. We note, in looking at the multivariate models by year, that the significance of gender fluctuates depending on the data year. This could have been the result of sampling dynamics and not indicative of changes in the population at large. A study asking similar questions in the same data year would have allowed for triangulation, but no such study was available. Finally, the main outcome variable is limited by its dichotomous nature and does not allow us to identify the specific type of provider respondents communicated online with within the previous 12 months. For example, a patient could have communicated electronically with a nurse or other member of the care team.

### Implications for Future Research

As the diffusion of this technology continues, future research related to online means of patient-provider communication should remain a focus. Future studies should try and garner a better sense of the frequency of communication and document the type of provider with whom the patient communicates to inform providers and health care organizations implementing new and additional means of online communication. Additionally, understanding use by disease type, including chronic illnesses, would be a useful addition to the literature. Although the adoption of these communication technologies is increasing among minority populations, another important area to explore is the quality of communication between minorities and their providers, and how it may influence, or be influenced by, the use of these technologies. Finally, with regards to the methodological approach utilized in this study, it is important to note that previously published studies are often not updated as new waves of data become available. HINTS data have been used in nearly 400 studies, and have yielded intriguing findings, but data updates to prior studies that use large datasets are infrequent. Perhaps this is due to instrument evolution across survey cycles that makes combining data across cycles challenging, but regardless, replication and extension of prior work is an area of research that warrants further attention.

### Conclusions

Despite initial challenges in implementation [6], the proportion of internet users communicating online with their health care providers has significantly increased since 2003. In addition, these trends are likely to continue with the enactment of the Health Information Technology for Economic and Clinical Health Act and Meaningful Use Stage 2 core objectives, which require providers to use secure electronic messaging to communicate with their patients. However, challenges still remain pertaining to the digital divide affecting individuals residing in nonmetropolitan areas and their access to the internet, making this group less likely to communicate online with their providers. Future research should continue to investigate patient-provider communication trends, specifically to gain an understanding of successful interventions that mitigate identified barriers from both provider and patient perspectives.

## Appendix

Multimedia Appendix 1 Analytical model and framework.

Multimedia Appendix 2 Odds of communicating online with a health care provider in the past 12 months by year.

## Abbreviations

CATI: Computer-assisted telephone interviewing
HINTS: Health Information National Trends Survey
OR: odds ratio
PHR: personal health record
USPS: United States Postal Service
