# Supplementary material for: Growth Dynamics of Patient-Provider Internet Communication: Trend Analysis Using the Health Information National Trends Survey (2003 to 2013)
Source: J Med Internet Res. 2018 Mar 29;20(3):e109. doi: 10.2196/jmir.7851 (PMC5897625; doi:10.2196/jmir.7851)
Supplement: Multimedia Appendix 1 [file jmir_v20i3e109_app1.pdf]

Appendix 1: Analytical Model and Framework

A three-part analytic process is used: 1) reanalysis, 2) close replication across years, and 3) trend analysis extension. During the reanalysis stage, the publicly available HINTS 1 (2003) and 2 (2005) data was used with the goal of identifying the precise analytic methodology used by in the original 2007 paper. The outcome was a binary response regarding if electronic communication with a provider occurred within the last year; the analysis used a logistic regression model.

$$\text{eleccommunication} = \alpha + \beta_1\text{age} + \beta_2\text{sex} + \beta_3\text{education} + \beta_4\text{income} + \beta_5\text{race} + \beta_6\text{insurance} + \beta_7\text{healthstatus} + \beta_8\text{cancerhistory} + \beta_9\text{metroarea} + \varepsilon$$

This stage served as a precursor to replication, ensuring that the model with additional data years is done with a high degree of fidelity to the analytic framework established as valid in the literature. In the close replication stage, we made minor alterations to the original analytic model to enable effective analysis across multiple data years (i.e., 2008, 2011, and 2013) in a manner that aligns with the original model as closely as possible while taking into account all available variables in each data year.

Finally, we engaged in extension - the analysis of the additional available data years, 2008, 2011, and 2013 - using the same analytic approach with the purpose of identifying trends over time. To explore these trends, we assign 2003 as our reference group while controlling for the subsequent data years of 2005, 2008, 2011, and 2013 (represented as datayr2, datayr3, datayr4, and datayr5, respectively, in our model).

$$\text{eleccommunication} = \alpha + \beta_1\text{age} + \beta_2\text{sex} + \beta_3\text{education} + \beta_4\text{income} + \beta_5\text{race} + \beta_6\text{insurance} + \beta_7\text{healthstatus} + \beta_8\text{cancerhistory} + \beta_9\text{metroarea} + \beta_{10}\text{datayr2} + \beta_{11}\text{datayr3} + \beta_{12}\text{datayr4} + \beta_{13}\text{datayr5}\ \varepsilon$$
