# Supplementary material for: Growth Dynamics of Patient-Provider Internet Communication: Trend Analysis Using the Health Information National Trends Survey (2003 to 2013)
Source: J Med Internet Res. 2018 Mar 29;20(3):e109. doi: 10.2196/jmir.7851 (PMC5897625; doi:10.2196/jmir.7851)
Supplement: Multimedia Appendix 2 [file jmir_v20i3e109_app2.pdf]

Appendix 2. Odds of Communicating Online with a Health Care Provider in the Past 12 Months by year

	HINTS 2003 (n=3,532) OR (95% CI)	*P* value	HINTS 2005 (n=2,651) OR (95% CI)	*P* value
**Age**	Reference: 18-34			
35-49	0.82 (0.54, 1.23)	0.319	0.79 (0.44, 1.41)	0.416
50-64	1.13 (0.76, 1.70)	0.537	0.79 (0.42, 1.48)	0.447
65-74	0.57 (0.26, 1.23)	0.146	0.48 (0.19, 1.22)	0.120
75 or older	1.08 (0.30, 3.88)	0.906	0.53 (0.16, 1.72)	0.285
**Gender**	Reference: Male			
Female	0.75 (0.55, 1.02)	0.063	1.47 (0.99, 2.18)	0.053
**Education**	Reference: Less than high school			
High school graduate	1.24 (0.34, 4.55)	0.746	0.56 (0.16, 1.99)	0.362
Some college	2.55 (0.71, 9.14)	0.148	0.88 (0.24, 3.31)	0.851
College graduate	**3.94 (1.13, 13.71)**	**0.032***	1.01 (0.28, 3.62)	0.983
**Annual Income (US $)**	Reference: < $10,000			
$10,000 to < $15,000	1.33 (0.25, 7.16)	0.736	0.40 (0.09, 1.79)	0.224
$15,000 to < $20,000	1.18 (0.22, 6.44)	0.844	0.21 (0.04, 1.26)	0.087
$20,000 to < $35,000	0.75 (0.18, 3.09)	0.680	0.53 (0.17, 1.69)	0.278
$35,000 to < $50,000	0.59 (0.14, 2.50)	0.465	0.45 (0.14, 1.50)	0.189
$50,000 to < $75,000	0.65 (0.15, 2.78)	0.557	0.54 (0.20, 1.43)	0.208
$75,000 or more	0.78 (0.19, 3.11)	0.718	0.78 (0.26, 2.34)	0.647
**Race/Ethnicity**	Reference: White			
Hispanic/ Latino	0.92 (0.48, 1.76)	0.803	0.54 (0.16, 1.80)	0.310
African American	0.90 (0.51, 1.58)	0.703	1.24 (0.62, 2.48)	0.536
Asian	0.81 (0.22, 2.97)	0.743	0.68 (0.14, 3.29)	0.623
Other	1.25 (0.43, 3.66)	0.676	1.55 (0.56, 4.29)	0.393
**Health Insurance**	Reference: Yes			
No	0.97 (0.51, 1.86)	0.933	0.98 (0.51, 1.88)	0.956
**Health Status**	Reference: Excellent			
Very Good	0.90 (0.54, 1.48)	0.661	1.33 (0.73, 2.42)	0.350
Good	1.07 (0.71, 1.62)	0.736	1.72 (0.95, 3.11)	0.072
Fair	1.51 (0.89, 2.58)	0.125	0.97 (0.45, 2.11)	0.947
Poor	0.97 (0.39, 2.40)	0.939	**2.83 (1.04, 7.68)**	**0.042***
**History of Cancer**	Reference: Yes			
No	0.74 (0.46, 1.18)	0.196	**0.52 (0.33, 0.82)**	**0.006†**
**Metropolitan Statistical Area**	Reference: Metro area County			
Non-metro area County	**0.62 (0.40, 0.95)**	**0.028***	0.74 (0.47, 1.16)	0.182

Key
*** = p < .05**
† = p < .01

Appendix 2. Odds of Communicating Online with a Health Care Provider in the Past 12 Months by year (continued)

	**HINTS 2008 (N=4,215)**		**HINTS 2011 (N=2,238)**		**HINTS 2013 (N=1,819)**	
	**OR (95% CI)**	***P* value**	**OR (95% CI)**	***P* value**	**OR (95% CI)**	**P value**
**Age**						
35-49	1.02 (0.72, 1.47)	0.893	0.75 (0.48, 1.16)	0.190	1.29 (0.78, 2.13)	0.317
50-64	0.80 (0.58, 1.10)	0.170	0.89 (0.59, 1.36)	0.591	1.08 (0.64, 1.81)	0.767
65-74	0.89 (0.60, 1.33)	0.572	0.94 (0.51, 1.75)	0.848	**0.56 (0.32, 0.99)**	**0.045***
75 or older	0.92 (0.43, 1.96)	0.831	1.81 (0.79, 4.12)	0.155	1.03 (0.46, 2.29)	0.949
**Gender**						
Female	1.06 (0.83, 1.36)	0.650	**1.55 (1.08, 2.22)**	**0.018***	**1.50 (1.02, 2.21)**	**0.040***
**Education**						
High school graduate	0.80 (0.24, 2.61)	0.704	1.26 (0.38, 4.18)	0.702	0.83 (0.20, 3.38)	0.792
Some college	0.82 (0.29, 2.33)	0.706	2.00 (0.57, 7.01)	0.272	2.08 (0.51, 8.48)	0.301
College graduate	1.32 (0.47, 3.69)	0.585	2.63 (0.75, 9.16)	0.127	2.39 (0.57, 10.06)	0.228
**Annual Income (US $)**						
$10,000 to < $15,000	1.21 (0.18, 8.06)	0.843	0.71 (0.12, 4.20)	0.699	2.37 (0.55, 10.16)	0.239
$15,000 to < $20,000	0.78 (0.19, 3.09)	0.713	1.09 (0.18, 6.75)	0.925	0.93 (0.22, 3.96)	0.919
$20,000 to < $35,000	0.68 (0.24, 1.97)	0.472	0.98 (0.15, 6.25)	0.986	1.06 (0.34, 3.25)	0.922
$35,000 to < $50,000	0.90 (0.32, 2.57)	0.847	1.32 (0.25, 6.86)	0.740	1.96 (0.58, 6.64)	0.273
$50,000 to < $75,000	0.94 (0.34, 2.58)	0.895	1.28 (0.25, 6.55)	0.766	2.38 (0.75, 7.53)	0.136
$75,000 or more	1.47 (0.54, 4.01)	0.449	1.62 (0.30, 8.63)	0.565	**3.10 (1.08, 8.85)**	**0.035***
**Race/Ethnicity**						
Hispanic/ Latino	1.25 (0.78, 2.03)	0.349	0.91 (0.48, 1.72)	0.765	1.21 (0.69, 2.11)	0.496
African American	1.13 (0.64, 2.01)	0.659	0.74 (0.40, 1.39)	0.347	1.04 (0.56, 1.93)	0.899
Asian	1.05 (0.54, 2.03)	0.888	1.45 (0.60, 3.52)	0.401	1.39 (0.71, 2.72)	0.332
Other	1.02 (0.48, 2.19)	0.959	1.70 (0.68, 4.29)	0.251	.98 (0.49, 1.94)	0.944
**Health Insurance**						
No	**0.47 (0.23, 0.95)**	**0.035***	0.50 (0.22, 1.10)	0.081	0.53 (0.26, 1.11)	0.091

Appendix 2. Odds of Communicating Online with a Health Care Provider in the Past 12 Months by year (continued)

**Health Status**						
Very Good	0.87 (0.58, 1.31)	0.512	0.74 (0.43, 1.28)	0.274	0.69 (0.40, 1.17)	0.165
Good	0.89 (0.61, 1.31)	0.553	1.07 (0.64, 1.77)	0.794	1.06 (0.61, 1.85)	0.824
Fair	1.38 (0.75, 2.57)	0.295	1.01 (0.35, 2.92)	0.988	1.08 (0.39, 2.97)	0.880
Poor	0.69 (0.25, 1.93)	0.468	1.29 (0.46, 3.65)	0.621	1.79 (0.27, 11.69)	0.537
**History of Cancer**						
Yes	0.79 (0.54, 1.16)	0.230	0.91 (0.59, 1.41)	0.669	**0.56 (0.32, 0.96)**	**0.036***
**Metropolitan Statistical Area**						
Non-metro area County	**0.72 (0.53, 0.96)**	**0.029***	**0.48 (0.28, 0.82)**	**0.008†**	0.63 (0.36, 1.08)	0.088

Key

*** = p < .05**
† = p < .01
